# Capillary bifurcations mechanically dissociate clusters of tumor cells

**DOI:** 10.1093/pnasnexus/pgag166

**Published:** 2026-05-14

**Authors:** Angelos Vrynas, Aisher Chen, Georgia Kontaxi, Sam H Au

**Affiliations:** Department of Bioengineering, Imperial College London, London SW7 2AZ, United Kingdom; Department of Bioengineering, Imperial College London, London SW7 2AZ, United Kingdom; Department of Bioengineering, Imperial College London, London SW7 2AZ, United Kingdom; Department of Bioengineering, Imperial College London, London SW7 2AZ, United Kingdom; Cancer Research UK Convergence Science Centre, London SW7 2AZ, United Kingdom

**Keywords:** circulating tumor cell clusters, cancer metastasis, microfluidics, capillary bifurcations, actin cytoskeleton

## Abstract

Circulating tumor cell (CTC) clusters are major drivers of cancer metastasis, possessing higher metastatic potential than equal numbers of single CTCs. While their ability to traverse linear capillary segments has been studied, their transit behaviors in capillary bifurcations remain poorly understood. We utilized live-cell imaging and microfluidic devices that replicate the diverse geometries of human capillary bifurcations to investigate the transit dynamics of breast cancer clusters. We demonstrate that clusters dissociate into smaller clusters and single cells and that this phenomenon is strongly influenced by geometric features of equal and small-sized daughter channels in capillary bifurcations. Moreover, inhibition of actin polymerization increases both the frequencies of cluster dissociation and cell lysis during cluster transit in capillary bifurcations. Overall, our findings suggest that capillary biomechanics and actin filament polymerization affect the integrity of CTC clusters.

Significance Statement Circulating tumor cell clusters must navigate through narrow capillary bifurcation networks to succeed in metastasis. We developed microfluidic models emulating the geometric heterogeneity of in vivo capillary bifurcation patterns to investigate cluster transit dynamics. Live imaging of clusters transiting in capillary bifurcations revealed their dissociation into smaller clusters or single cells, enhanced particularly by equal and small-sized daughter channels or cellular F-actin depolymerization. Moreover, disruption of F-actin led to an increase in cell lysis in cells within clusters downstream of capillary bifurcations. These findings expand our understanding of the integrity and transit behavior of tumor cell clusters during metastasis.

## Introduction

Clusters of circulating tumor cells (CTC clusters) are significantly more capable of seeding metastases compared with equal numbers of individual CTCs, with some studies suggesting they are up to 50-fold more potent (1). This potency has been attributed to their resistance to anchorage-dependent apoptosis (anoikis), greater proliferation rates, immune evasion capabilities, gain of stemness, and plasticity (1–6). Given their increased metastatic potency over single CTCs, it is critical to understand how CTC clusters navigate through the circulation.

We previously demonstrated that CTC clusters, even those containing over 20 cells, are capable of transiting through linear segments of capillary constrictions by unfolding into single-file chains (7, 8). However, the architecture of the arterial microvasculature is not a series of linear channels but is better described as a fractal network of progressively narrowing bifurcations. These microvasculature bifurcations mechanically impede and cause the deformation of CTCs in transit (9, 10), and the navigation of breast cancer cells through these bifurcations may be indicative of their metastatic potential (11, 12). Szczerba et al. previously isolated clusters from both peripheral veins of mouse models of breast cancer and tumor-draining vessels that precede capillaries. Interestingly, clusters recovered from vessels downstream of capillaries were on average smaller (i.e. comprised of fewer cells) than those recovered from vessels preceding capillaries (13). One explanation is that clusters with higher cell counts tend to arrest in capillaries, whereas clusters with low cell counts and single CTCs have higher probabilities of escaping capillary entrapment. However, we hypothesize that there exists an additional phenomenon that can explain the increased prevalence of clusters comprised of fewer cells, isolated downstream of capillary beds.

In this work, we used microfluidic devices we designed to mimic the diverse sizes and degrees of symmetric and angular geometries present in human microvascular bifurcations to observe the transit of cancer clusters through capillary bifurcations. We observed that capillary-scale bifurcations promoted the dissociation of clusters into smaller clusters and single cells during transit. Live-cell imaging revealed that narrower capillary bifurcations and equal daughter branch sizes alongside F-actin depolymerization promote cluster dissociation. This dissociation of tumor clusters may play a critical role in the metastatic process by dynamically altering the organization, survival, and behavior of CTCs.

## Results

### Tumor cell clusters dissociate in capillary bifurcations

**Figure 1:**
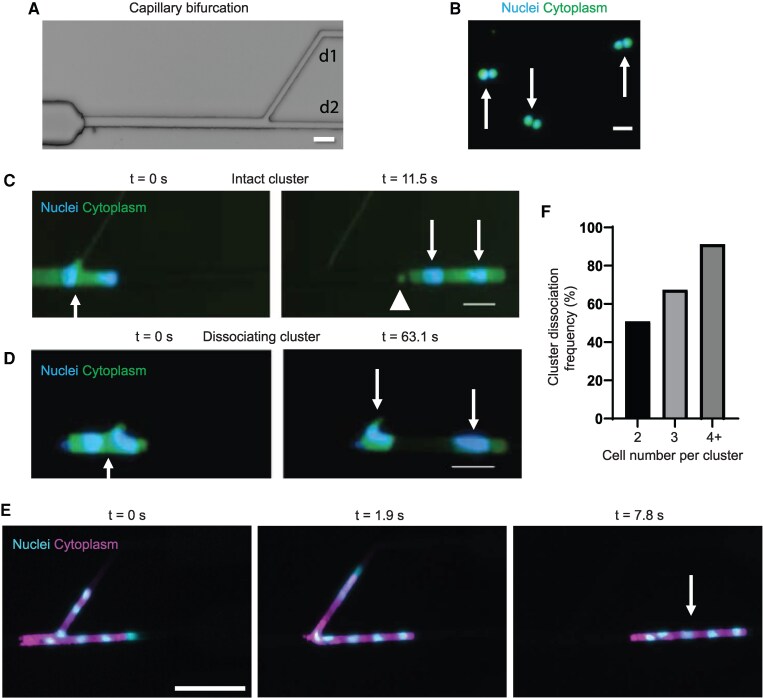
Cluster dissociation in capillary bifurcations. A) Brightfield image of an engineered microfluidic capillary bifurcation device (ENA_7/7). Scale bar: 20 μm. B) Fluorescent image of three two-cell clusters tagged with green cell tracker (cytoplasm) and Hoechst (nuclei). Arrows indicate clusters. Scale bar: 20 μm. C) Timelapse series of a two-cell cluster (arrows) remaining intact while transiting a capillary bifurcation (ENA_7/7). A shearosome is also observed forming after cluster transit in bifurcation (white arrowhead). Scale bar: 40 μm. D) Timelapse series of a two-cell cluster dissociating (arrows) while transiting a capillary bifurcation (ENA_7/7). Arrows indicate original cluster and dissociated single cells. Scale bar: 40 μm. E) Timelapse series of a seven-cell cluster transiting through a capillary bifurcation (ENA_7/7). Far-right image shows a five-cell cluster (arrow) that dissociated from the original cluster, and other fragments from the original cluster are out of frame. Cytoplasm and nuclei were stained with green cell tracker (shown as magenta) and Hoechst (shown as cyan). Scale bar: 100 μm. F) Dissociation frequency of doublets, triplets, and quadruplets+ in capillary bifurcations (EWA, EWS, UWA, UWS, UNA_5/9, and ENA_7/7).

To study the behavior of cancer clusters at bifurcations, we used microfluidic bifurcation devices (Fig. 1A) that mimic the diverse geometries of human microvasculature, alongside a nonbifurcating (“linear”) (7 μm) and a nonconstricted (20 μm) control device that we previously developed (see also Table S1 for devices nomenclature and Fig. S1) (14). We introduced clusters of MDA-MB 231 breast cancer cells into these capillary bifurcation microfluidic devices (Fig. 1B) to observe their transit under physiological pressure (30 cm H_2_O).

We observed clusters entering capillary bifurcations via live-cell imaging. Seventy-six percent were two-cell (doublets) clusters, while 16 and 8% consisted of three-cell (triplets) clusters or four or greater (quadruplet+ or 4+) clusters, respectively (Fig. S2A and B). These distributions were consistent with previous reports on CTC cluster distributions from patient samples (1, 13).

We then observed the behavior of clusters transiting through capillary bifurcations. Some clusters passed without any cells dissociating from the clusters (intact clusters) (Fig. 1C). Other clusters dissociated into smaller clusters and/or single cells (dissociating clusters; Fig. 1D and Movies S1–S4). We observed dissociating clusters that contained as many as seven cells (Fig. 1E and Movie S1). Cluster dissociation was observed in all tested geometric variations of daughter branches, including those with equal/unequal branch sizes, narrow/wide angle branching, and symmetry/asymmetry pathing (Movies S1–S4). We also observed the frequent biogenesis of shearosomes, shear-derived large extracellular vesicles that we recently characterized (14) (Fig. 1C, Movies S3 and S4). We then quantified the dissociation frequency of clusters: 51% of doublets, 67% of triplets, and 91% of quadruplets+ clusters experienced at least one dissociation event during transit (Figs. 1F and S2C). Based on the frequency of dissociation of doublet clusters, we then performed a theoretical analysis of the expected probability of dissociation in clusters containing three or more cells (Table S2). Our data suggest that the probabilities of dissociation for triplets, quadruplets, and larger clusters grow in line with the number of intercellular junctions within a cluster as predicted by our probabilistic analysis that assumes each junction behaves independently of others (Table S2).

### Pathing of tumor cell clusters through capillary bifurcations

**Figure 2:**
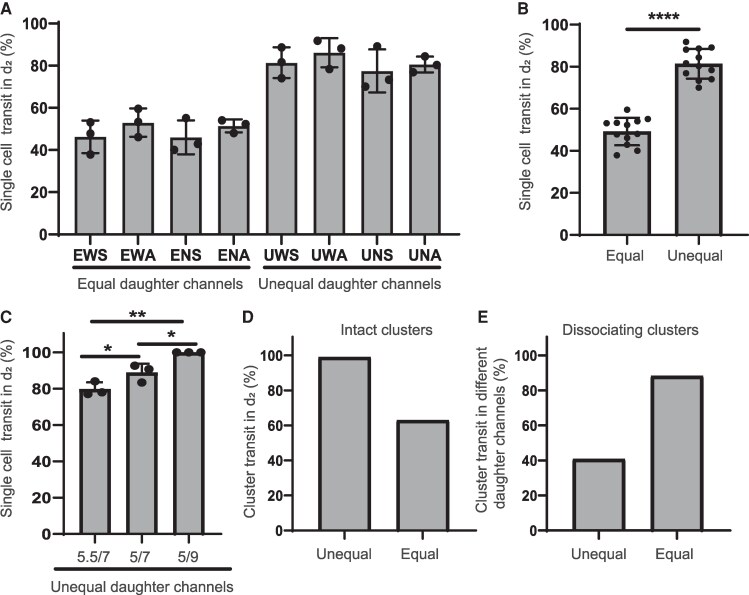
Cluster and single cells preferably exit from lower resistance daughter channels. A) Percentage of single MDA-MB231 cells recorded to move through *d*_2_ daughter branch channel, quantified for eight distinct capillary bifurcation variants (*n* = 3). B) Percentage of single MDA-MB231 cells recorded to move through *d*_2_ daughter branch channel in equal (*n* = 12) or unequal (*n* = 12) bifurcations, data pooled from variants in A. C) Percentage of single MDA-MB231 cells recorded to move through *d*_2_ daughter branch channel, quantified for three distinct unequal capillary bifurcation variants (UNA_5.5/7, UNA_5/7, and UNA_5/9; *n* = 3). D) Percentage of intact clusters that moved through *d*_2_ daughter branch channel in unequal or equal capillary bifurcation variants. E) Percentage of dissociating clusters that moved through different daughter branch channels in unequal or equal capillary bifurcation variants.

Before exploring how the dissociated components of clusters pathed through bifurcations, we first validated that single cells and intact clusters pathed as expected, as previous work suggested for single cells in bifurcations (11). As anticipated, when transiting through bifurcations with equal-sized daughter channels, single cells pathed approximately equally through daughter branches compared with unequal-sized daughter channels (Fig. 2A, B and S3A). The angle of the bifurcation did not affect pathing (Fig. S3B). In bifurcations with unequal daughter channel sizes, the majority of single cells showed greater preference for the larger daughter branch as the disparities in daughter branch diameters grew (Figs. 2A, C and S3A). Similar observations were found for intact clusters transiting through unequal and equal daughter branch size bifurcations (Fig. 2D).

We also sought to investigate the influence of daughter channel symmetry on pathing preference. In symmetric designs, daughter channels branched off at identical angles from parental branches. In contrast, asymmetric designs featured one daughter channel that continued linearly in the same direction as parental branches while the other branched off at angle (Fig. S1 and Table S1). Our results indicate that daughter branch symmetry did not lead to statistically significant differences in pathing preference (Figs. 2A and S3C). These results suggest that at the observed flow conditions, pathing is governed primarily by hydrodynamic resistance and pressure gradients rather than inertial momentum, consistent with low Reynolds number laminar flow regimes.

We then investigated the pathing of dissociating clusters (Fig. 1D). As expected, the components of 100% of dissociating clusters moved through the larger *d*_2_ daughter channel in unequal bifurcations, but in equal bifurcations, dissociating clusters pathed in either daughter branch nearly equally (Fig. S3D). We then explored the percentage of the components from dissociating clusters that preferentially pathed through the different daughter channels of a bifurcation. In equal bifurcations, the components of 88% dissociating clusters moved through different daughter branches, whereas in unequal bifurcations, this was the case for only 41% of clusters (Fig. 2E). Altogether, these findings suggest that clusters and their components preferentially exit bifurcations through daughter branches of lower hydrodynamic resistance.

### Small- and equal-sized capillary bifurcations promote cluster dissociation

**Figure 3:**
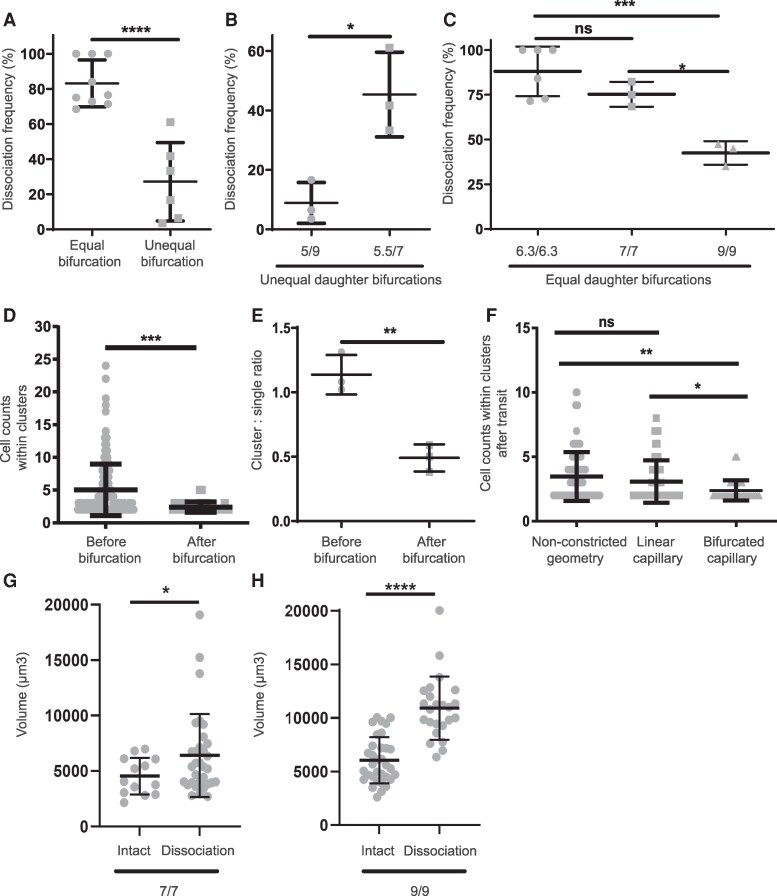
Features of capillary bifurcations that promote cluster dissociation. A) Dissociation frequency of all clusters during their transit in equal (*n* = 9) or unequal (*n* = 6) capillary bifurcations. B) Dissociation frequency of all clusters during their transit in unequal capillary bifurcations (UNA_5/9 and UNA_5.5/7; *n* = 3). C) Dissociation frequency of all clusters during their transit in equal capillary bifurcations (ENA_6.3/6.3 [*n* = 6] and ENA_7/7 [*n* = 3] and ENA_9/9 [*n* = 3]). D) Cell counts of clusters before (*n* = 182) and postcapillary bifurcation collection (*n* = 33). E) Cluster: single-cell ratio before and postcapillary bifurcation collection (*n* = 3). F) Comparison of cell counts of clusters after collection from capillary bifurcations (*n* = 33), linear capillaries (*n* = 53), and nonconstricted geometry (*n* = 106). G) Volume of intact (*n* = 13) or dissociating clusters (*n* = 32) in equal capillary bifurcations of 7 μm. H) Volume of intact (*n* = 33) or dissociating clusters (*n* = 24) in equal capillary bifurcations of 9 μm.

We compared how features of capillary bifurcation variants affected dissociation frequency. First, we compared dissociation frequencies of clusters between equal and unequal daughter branches. Clusters transiting through equal bifurcations experienced a statistically significant increase in the mean dissociation frequency vs. unequal counterparts (83 ± 13 vs. 27 ± 22%, *P* < 0.0001; Fig. 3A). Similar dissociation frequencies were calculated for doublets only (Fig. S4A).

Then, we wondered whether size differences between daughter branches also affected cluster dissociation frequencies. During transit through unequal-sized daughter branches, a smaller difference between the two daughter branches significantly increased the mean dissociation frequency of all clusters (e.g. UNA_5.5/7: 45.3 ± 14% vs. UNA_5/9: 8.9 ± 7%, *P* < 0.05; Fig. 3B) or doublets only (Fig. S4B). In equal-sized daughter branches, the mean cluster dissociation frequency increased with decreasing daughter branch sizes (Fig. 3C). For example, capillary bifurcations of 7 μm led to nearly a 2-fold higher dissociation frequency than their 9 μm counterparts for all clusters (73.3 ± 4 vs. 42.4 ± 6%, *P* < 0.05; Fig. 3C) and nearly 20-fold higher for doublets (Fig. S4C). These findings suggest that equal bifurcations, small constriction sizes and small size differences between daughter branches promote cluster dissociation.

We then explored the importance of bifurcations and constricted transit on cluster cohesion during transit. There was a significant decrease in the mean number of cells (before: 5 ± 3.9 vs. after: 2.4 ± 0.8, *P* < 0.0005; Fig. 3D) and cluster:single-cell ratios (before: 1.1 ± 0.1 vs. after: 0.5 ± 0.1, *P* < 0.005; Fig. 3E) due to transit through capillary bifurcations that suggests frequent dissociation events consistent with our observations of real-time dissociation (Fig. 2C). We also noted reductions in the mean number of cells within clusters after transit in nonconstricted geometries (Fig. S5A) and linear capillaries (Fig. S5B) but at significantly smaller magnitudes compared with capillary bifurcations (Fig. 3F). Moreover, after transiting capillary bifurcations, no clusters contained more than five cells, while nonconstricted and linear geometries contained up to 10 and eight cells, respectively (Fig. 3F). Overall, these findings support the notion that mechanical features of capillary bifurcations affect the dissociation of clusters.

### Cluster volume influences dissociation probability

Using our live-cell imaging analysis, we calculated the total cytoplasmic volume of both dissociating clusters (calculated prior to dissociation) and intact clusters, for comparison. Dissociating clusters had significantly higher volumes compared with intact clusters when transited through both 7 and 9 μm capillary bifurcations (Fig. 3G and H), suggesting a link between cluster size and dissociation. The volumes of dissociating clusters were 41 and 80% larger than intact clusters transiting through equal capillary bifurcations of 7 and 9 μm (Fig. 3G and H), respectively, highlighting the influence of the ratio of cluster to capillary sizes on dissociation. Unsurprisingly, the mean volumes of both intact and dissociating clusters were higher in equal capillary bifurcations of 9 μm compared with the smaller 7 μm ones (Fig. S6), suggesting that increases in capillary size also increase the volumetric thresholds for dissociation.

### Actin polymerization modulates cluster dissociation frequency and viability

**Figure 4:**
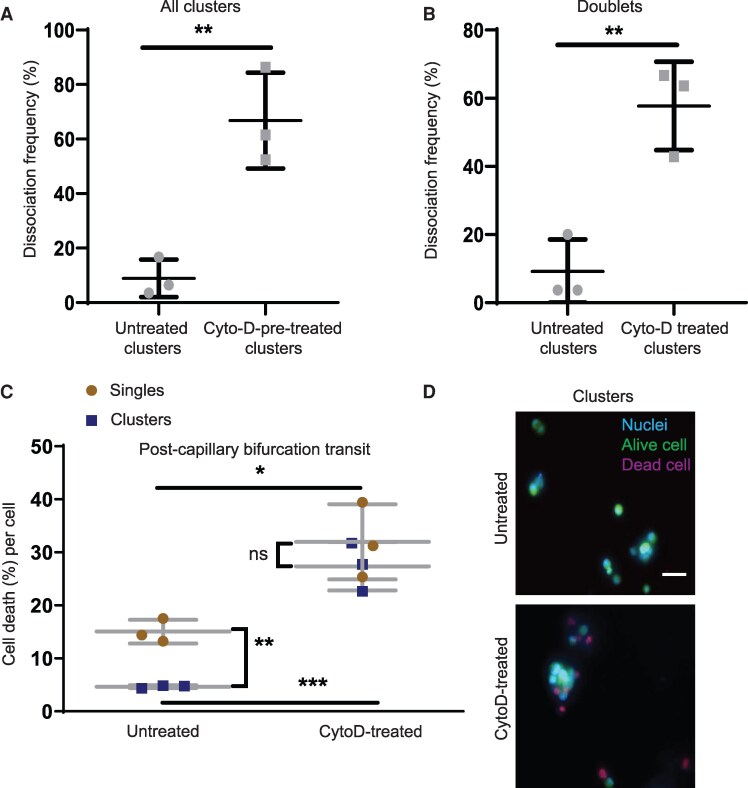
Cyto-D promotes cluster dissociation and cell death in capillary bifurcations. A) Dissociation frequency of untreated or Cyto-D-pretreated clusters in capillary bifurcation UNA_5/9 (*n* = 3). B) Dissociation frequency of untreated or Cyto-D-pretreated doublets in capillary bifurcation UNA_5/9 (*n* = 3). C) Percentage of cell death of untreated or Cyto-D-pretreated clusters or single cells postcollection from capillary bifurcation (*n* = 3). D) Fluorescent images of untreated (top) and Cyto-D-pretreated clusters after collection from capillary bifurcations. Alive cells were stained with green cell tracker (green) and dead cells with PI (red). Nuclei were stained with Hoechst (blue). Scale bar: 50 μm.

Our previous work suggested that the strength of intercellular adhesions influences the integrity of clusters during transit through capillaries (7). Given the importance of cytoskeletal F-actin to intercellular adhesion within clusters (15, 16), we examined whether chemical inhibition of F-actin polymerization could alter dissociation frequencies. To this end, we introduced untreated or cytochalasin D (Cyto-D)-pretreated clusters separately into unequal capillary bifurcations (UNA_5/9). This geometry was chosen because it led to a lower baseline dissociation frequency (Fig. 2B). Treatment of clusters with Cyto-D led to an approximate 7.5-fold higher mean dissociation frequency compared with untreated clusters within capillary bifurcations (66.7 ± 17% vs. 8.9 ± 7%, *P* < 0.05) (Fig. 4A). Taking into account the influence of cluster size on dissociation frequency (Fig. 1E), we also found a 6.5-fold higher mean dissociation frequency in capillary bifurcations by doublets treated with Cyto-D (57.7 ± 13 vs. 9.1 ± 9%, *P* < 0.05; Fig. 4B).

To explore whether assembling into clusters provided a protective effect for cells transiting through bifurcations, we observed single and clustered cells during transit through capillary bifurcations or nonconstricted devices (shear only controls). Cells within clusters had better survival after transiting both capillary bifurcations (Fig. 4C and D) and nonconstricted geometries (Fig. S7A and B).

We next examined the role of Cyto-D on cell viability during transit through capillary bifurcations. After transit through capillary bifurcations, Cyto-D induced a 2-fold higher rate of cell death in single cells (32 ± 7 vs. 15 ± 2%, *P* < 0.005; Fig. 4C) and a 6-fold higher rate in cells within clusters (27.3 ± 5 vs. 4.6 ± 0.2%, *P* < 0.005; Fig. 4C and D). While untreated cells within clusters survive at significantly higher rates compared with single cells, treatment with Cyto-D led to indistinguishable (*P* > 0.05) survival rates between clusters and single cells (Fig. 4C). Cyto-D treatment on cultures of clusters under static conditions did not significantly increase cell death (Fig. S7C). Treatment with Cyto-D did not affect the transit speed of intact or dissociating clusters (Fig. S8A and B) nor the dissociation time in capillary bifurcations (Fig. S8C and D), but we did observe an increase in lysis compared with untreated clusters (Fig. S8E).

Cyto-D also increased the rates of cell death in nonconstricted controls both for single cells and clusters (Fig. S7A and B), but at lower levels than those we quantified in capillary bifurcations. These findings suggest that both cluster dissociation frequency and cell lysis under constricted transit through bifurcations are enhanced upon inhibition of F-actin polymerization. However, since F-actin modulation may influence numerous co-interacting biomechanical properties, it is difficult to distinguish between the specific contributions of weakened intercellular adhesion, altered cellular deformability, or increased mechanical fragility on cluster stability and viability during constricted transit.

## Discussion

Our data suggest that tumor cell clusters transiting through capillary bifurcations at physiological pressures can dissociate into smaller clusters and single cells due to mechanical forces applied to these cellular aggregates within constrictions. Dissociation appears to occur more frequently when several factors are present, including (a) shear, (b) small- and equal-sized capillary bifurcations, (c) larger clusters containing greater numbers of cells, (d) small size differences between the daughter channels in bifurcations, and (e) F-actin integrity is compromised. The observation that F-actin regulates dissociation at bifurcations suggests that cytoskeletal integrity is a key regulator of cluster behavior. However, the effects of Cyto-D and F-actin polymerization inhibition are multifaceted and, therefore, our findings suggest that actin integrity broadly modulates cluster survival and integrity at bifurcations. Future studies are required to isolate whether this is due to other changes in cell-intrinsic properties, such as membrane fluidity, nuclear stiffness, or the direct strength of intercellular adhesions.

Our capillary bifurcation devices were engineered to account for the geometric diversity present in human capillaries. For instance, we designed daughter branch microchannels of widths ranging from 5.5 to 9 microns to match capillary diameters that range from 5 to 10 μm, as well as daughter branch dimensions, symmetry, and angle microfluidic devices were also operated at hydrostatic pressures equivalent to those found across human capillaries. These models provided us with mechanistic insights into the parameters that drive cluster dissociation. More sophisticated experiments are needed, however, to fully understand the dynamics of tumor cell cluster dissociation in humans. For example, while we model the behavior of cluster transiting through single bifurcations, clusters encounter numerous fractally bifurcating and narrowing vessels in the microvasculature that may lead to more complex dissociation kinetics not studied here. While the microchannels used in this work have precise size tolerances, biomaterial modifications could be made to better mimic in vivo deformations and friction at cell-wall interfaces to increase the physiological relevance of our models (17). Furthermore, endothelial cell (18) and immune cell (19) interactions with circulating tumor cells may also modulate cluster dissociation kinetics, and thus, future models should be engineered to incorporate these elements. Previous studies have highlighted the prevalence of two- and three-cell clusters and generally smaller clusters (13, 20, 21) among single cells isolated from peripheral veins as opposed to tumor-draining vessels, e.g. before capillary transit (13). One explanation is the more frequent arrest of larger clusters in in vivo capillaries. However, our findings suggest that certain features in capillary bifurcations, e.g. small capillary size, can promote dissociation of clusters, providing a supplemental explanation of the above observations.

Our findings also reveal that the frequency that a given cluster undergoes at least one dissociation event is affected by the number of cells in a given cluster. To conduct this analysis, we used our experimentally observed dissociation rate of two clusters to generate a probabilistic prediction for larger clusters, assuming that dissociation frequency would grow with the number of junctions within a cluster traveling in single file. Our experimental data are in relatively close agreement with this our predictions (Table S2). This suggests that when clusters encounter a bifurcation, each intercellular junction within a cluster has a probability of being broken and that these probabilities are not affected by the integrity of other junctions or transit order. Deviation from our theoretically calculated dissociation frequencies may be partially explained by deviation from any of our assumptions: i.e. that (a) clusters transited through constrictions in single file, (b) intercellular adhesions between cells in doublets are identical to those between triplet, quadruplets, and higher order clusters, and (c) the maintenance or disruption of adhesions in one part of a cluster does not alter the adhesive strengths of other junctions. It should also be noted that dissociation frequency is highly context dependent. Dissociation probabilities likely differ depending upon geometry, constriction sizes, and, importantly, the adhesive nature of cells within a cluster. For instance, the MDA-MB-231 triple-negative breast cancer tumor cell clusters used in this work have a mesenchymal phenotype, and, therefore, their dissociation characteristics will differ from clusters comprised of more tightly cohesive cells, such as those with epithelial phenotypes. Context is therefore a key in predicting cluster integrity at microvascular bifurcations and is inherently heterogeneous due to different adhesive characteristics among cancer subtypes, patient-to-patient variability, and the degree of intratumoral heterogeneity from which CTCs are derived.

While there is substantial evidence of the greater metastatic potential of CTC clusters compared with equal numbers of individual CTCs (1, 2, 8), the relative potential of larger CTC clusters consisting of more cells compared with those dissociated into more numerous yet smaller clusters consisting of fewer cells remains unclear. Some explanations for greater CTC cluster survival and metastasis, such as the inhibition of anoikis due to the maintenance of cell–cell adhesion within clusters, are not likely to be appreciably weakened in smaller clusters. In these cases, dissociation into more numerous clusters may provide additional opportunities for successful metastasis in target organs. Other explanations, however, such as the shielding of cells from vascular shear stress within clusters, may be more complex. For instance, the data in this work suggest that cells within clusters retained higher levels of viability after transit in either capillary bifurcations or nonconstricted shear controls. These findings are consistent with previous work highlighting that single cells can in fact be vulnerable to shear stress and forces in capillary (22–27). However, the impact of shear stress shielding in larger vs. smaller clusters is unclear. Smaller clusters inherently have greater surface area:volume ratios, and thus, a greater proportion of cells within each cluster are exposed to the harmful effects of shear in a nonlinear manner. Further studies are therefore needed to understand how bifurcation-induced cluster dissociation affects metastasis and whether promoting or inhibiting cluster dissociation, such as through the interference of the actin cytoskeletal network by Cyto-D, helps or hampers clusters of disparate sizes to succeed at metastasis.

## Materials and methods

### Cell culture and reagents

MDA-MB-231 breast cancer cell line was purchased from ATCC, and all reagents were purchased from Sigma (United Kingdom), unless otherwise noted. Cells were grown in StableCell-Dulbecco's modified Eagles medium (DMEM) media, supplemented with 10% (v/v) fetal bovine serum and 1% (v/v) penicillin/streptomycin. Cells were maintained at 37 °C/5% CO_2_ conditions and subcultured every 2–3 days. Single cells were grown initially either in confluent 48-well plates (Corning, United Kingdom) or 25 cm^2^ tissue culture flasks (Corning). Then, cells were washed briefly with phosphate-buffered saline (PBS) and detached by adding trypsin (0.25% v/v) for 5 min at 37 °C/5% CO_2_. DMEM media–containing serum was then added to cell suspension to inactivate trypsin and centrifuged at 180*×g* for 5 min. The cell pellet was either resuspended with fresh media and transferred to new T25 flasks or was stained directly for downstream experiments. The staining solution contained 1 mL of DMEM, Calcein-AM (Thermo Fisher Scientific, United Kingdom) diluted down to 5 μM and Hoechst 33342 diluted down to 16.2 μM. The staining solution was added to the cells for 15 min at 37 °C/5% CO_2_.

### Cluster preparation

Wells of a 96-well plate were initially pretreated with 1% Pluronic F-127 (Thermo Fisher Scientific) for 1 h at room temperature. Single MDA-MB 231 tumor cells were harvested as above. Cells were enumerated via a hemocytometer, and 100 μL of cell suspension (10,000 cells/100 μL) was added to separate wells of a round 96-well plate and left for 24 h to grow as spheroids. After the 24 h, the staining solution for labeling cells (as prepared above) was added directly to the wells. After 15 min incubation, the formed large aggregates/spheroids were mechanically dissociated by resuspension eight to 10 times to dissociate them in clusters.

### Design and fabrication of microfluidic devices

We previously generated 13 different capillary bifurcations (14) by varying channel width, symmetry (daughter channels branching off at the same angle from parent channel), and angle of daughter channels (Table S1 and Fig. S1) to emulate the geometric diversity present in the in vivo microvasculature. We further designed two control geometries, as outlined in Supplementary Table S1, termed as nonbifurcated (linear) and nonconstricted. Branch geometries were designed according to Murray's law


(1) $$d_{0}^{3}=d_{1}^{3}+d_{2}^{3},$$

where *d*_0_ represents the effective diameter of the parent channel and *d*_1_ and *d*_2_ represent the effective diameters of the daughter branched channels.

Moreover, for each diameter in Eq. 1, we used Eq. 2 to extrapolate the effective diameter of channels <10 μm.


(2) $$d_{e}=1.3\times (a\times b{)}^{0.625}/(a+b{)}^{0.25},$$

where *a* and *b* define the width and height of each channel.

Devices were fabricated using photolithography and soft lithography as previously described (14). Briefly, we sterilized silicon wafers (MSE Supplies, Germany) with 99% isopropanole (IPA) and subsequently 100% acetone. Wafers were treated with oxygen plasma (Harrick plasma cleaner) at 0.5 Tor O_2_ at 30 W for 1 min and then spin coated with a GM1050 SU-8-negative photoresist (Gersteltec, Switzerland) before transferring SU-8-coated wafers to a UV-KUB 3 mask aligner (KLOE, France), where they were exposed for UV exposure through chrome glass masks (Microlitho, United Kingdom). After baking, wafers were submerged in SU-8 developer (Kayaku Advanced Materials, United States) for 5 min. We used an optical surface profilometer (Filmetrics, United States) to verify that the height of the devices was within 10% of set points: constricted capillaries (bifurcation and linear), 7 μm and nonconstricted geometries, 20 μm. Wafers were transferred to petri dishes and secured with tape.

Then, Sylgard 184 polydimethylsiloxane (PDMS) prepolymer was mixed with its crosslinker (Dow Corning) at a 10:1 w/w ratio and poured onto the taped silicon master wafers, degassed under vacuum for 30 min to remove bubbles, and left to polymerize overnight at 65 °C in an oven. After cutting out the polymerized PDMS with a scalpel, we punched inlets and outlets on the PDMS replicas with a 4- and 2-mm biopsy punch (IHC World, United States), respectively. To enhance PDMS attachment to glass slides, we treated both with oxygen plasma (0.5 mm Tor, 30 W, 1 min/Blackhole, France) before bonding PDMS replicas to glass microscope slides. We heated the PDMS microfluidic devices for 10 min at 95 °C to stabilize the bonding.

To prepare our microfluidic devices for downstream cell work, we first were sterilized and primed channels 70% v/v ethanol. We then washed the channels with 1× PBS to remove ethanol. Finally, we flushed the devices with 3% bovine serum albumin (w/v) solution to inhibit adhesion of cells to microchannel surfaces.

### Fluorescent microscopy

Generally, for the acquisition of static fluorescent and brightfield images of single cells and clusters and live imaging during their transit in microfluidic devices, we used a Nikon Eclipse Ti2 multifluorescent inverted microscope with an Okolab incubated stage (Nikon, United States). Well plates or microfluidic devices were loaded onto the microscope stage and imaged as required. Excitation/emission settings for imaging cytoplasm (green cell tracker) and nuclei (Hoechst) were used at 488/528 and 350/461 nm.

### Cluster transit experiments

Capillary designs and their fabricated microfluidic variants mimicking human microvessels were prepared, as published previously (14). Briefly, for transit experiments, 1 m long tubing of 2 mm diameter (Qosina, United States), prefilled with PBS, was used to prime the outlet of microfluidic devices and a 10-mL syringe through a 22-G needle (Needlez, United Kingdom; henceforth termed as the syringe-tubing setup). The tubing/syringe setup was prefilled with 2 mL PBS, the plungers were removed, and the free ends of the tubing were connected to the outlets of the devices. Thirty microliters of cluster suspensions (10,000 cells/100 μL) were added to the inlet of microfluidic devices. To generate pressures occurring physiologically in capillaries, the syringe was lowered at 30 cm below the level of the microfluidic device. Live imaging of cluster transit was recorded at high speed (3 frames/s), and the images were compiled together to form a video series. Alternatively, cluster suspensions were added in separate microfluidic devices, transited and collected by emptying the liquid content of syringes directly in wells of a 96-well plate for downstream experiments.

### Cell treatment

Single or clustered cells were treated with an F-actin polymerization inhibitor, Cyto-D at a final working concentration of 10 μM in media for 3 h at 37 °C/5% CO_2_ before further analysis or introduction into devices. Cyto-D-pretreated clusters were imaged either (a) during transit in capillary bifurcations or (b) posttransit from capillary bifurcations or nonconstricted geometries.

### Analysis of cluster transit and dissociation

Analyses of cluster transit through capillary bifurcations were conducted manually using the Nikon microscope. Via live imaging, cluster dissociation was verified when cells or smaller clusters detached from the main cluster unit, indicated by a distance between Hoechst- and Calcein-positive cells. Dissociation frequency was extrapolated by dividing the number of dissociating clusters (at least one dissociation event) by the overall number of transiting clusters through various capillary bifurcation devices. To minimize the possibility that colliding single cells would appear as clusters, incoming clusters were tracked and monitored before their encounter with the capillary bifurcation. Volumes of clusters and individual cells within clusters transiting in capillary bifurcations were calculated from photomicrographs as previously described (7). Cells were positive for lysis during live imaging by observation of loss of cytoplasmic dye.

Theoretical analyses of probabilities of cluster dissociation frequencies were calculated based on the experimental calculations of doublet dissociation frequency. To simplify analyses, clusters were assumed to be transiting in single file, so that only two cells within a cluster are connected at any time, i.e. a triplet has *n* = 2 possible dissociation events and a quadruplet has *n* = 3. Thus, to calculate the probability of at least one dissociation event *P*(at least one), we used the following formula 1, where *P*(none) is the probability of no dissociation event happening, *P*(*A*) is the probability of a dissociation event *A* happening between two cells and *P*(*A*′) is the probability of event *A* not happening, defined as *P*(*A*′) = 1 − *P*(*A*), where *P*(*A*) was taken as 0.51 from observations of two-cell cluster dissociation frequencies.


(3) $$P(\mathrm{at}\;\mathrm{least}\;\mathrm{one})=1-P(\mathrm{none})=1-P(A^{\prime }{)}^{n}=1-(1-P(A){)}^{n}.$$

### Viability assay

Live/dead viability assay was performed on both single and clustered cells. The live/dead stain mixture was prepared by diluting Calcein-AM (live-cell marker) at 5 μM, Hoechst 33342 (nuclei) at 16.2 μM and propidium iodide (PI; dead cell marker) 1 μg/mL, in serum-free DMEM media. The staining solution was added to single or clustered cells that were collected posttransit from either capillary bifurcations or nonconstricted geometries. For control experiments (no flow), cell suspensions were introduced into microfluidic devices (inlets), but without any flow. Where required, cells were pretreated with Cyto-D, as described above. Cells were verified as alive, if they were Calcein positive, Hoechst positive but PI negative, whereas they were verified as dead, if they were Calcein negative, Hoechst -positive, and PI positive. Cell death within clusters was quantified on a per cell basis.

### Analysis of collected cells postdevice transit

Clustered cell suspensions were stained, added in microfluidic devices, and collected posttransit from capillary bifurcations, linear capillaries, and nonconstricted geometries, as elaborated above. Fluorescent images of clusters were obtained before and after transit, using the Nikon microscope. By analyzing these images, the number of clusters, the number of single cells, and the number of cells per cluster were quantified. Alternatively, unstained single or clustered cell suspensions were added either in capillary bifurcations or nonconstricted geometries, collected, and stained for viability, as described previously.

### Graphs and statistical analyses

All experiments were performed at least three times. Collected and analyzed data were presented as mean ± SD. The relevant graphs were plotted and analyzed statistically via the GraphPad Prism (version 9.0) software. Statistical comparison between two experimental groups was performed by applying the two-tailed Student's t test. For statistical comparison among three or more experimental groups, one-way ANOVA (Bonferonni’s or Tukey's multiple comparisons) was applied with a 95% CI. Statistical differences between experimental groups that were not significant were denoted as ns and presented *P* values ≥0.05. Alternatively, for *P* values <0.05, statistical significance was achieved and described as (a) significant (0.01 to 0.05, single asterisk*), (b) very significant (0.001 to 0.01, double asterisk **), and (c) extremely significant (0.0001 to 0.001, triple asterisk *** or <0.0001, quadruple asterisk ****). Illustrations were prepared using BioRender software.

## Supplementary Material

pgag166_Supplementary_Data

## Data Availability

Raw data supporting the findings of this study are available in the Supplementary material and at https://doi.org/10.5281/zenodo.20120169.
