# Supplementary material for: Capillary bifurcations mechanically dissociate clusters of tumor cells
Source: PNAS Nexus. 2026 May 14;5(5):pgag166. doi: 10.1093/pnasnexus/pgag166 (PMC13197843; doi:10.1093/pnasnexus/pgag166)
Supplement: pgag166_Supplementary_Data [file pgag166_supplementary_data.zip › PNASNEXUS-PNASNEXUS-2025-01887RR-s01.docx]

**
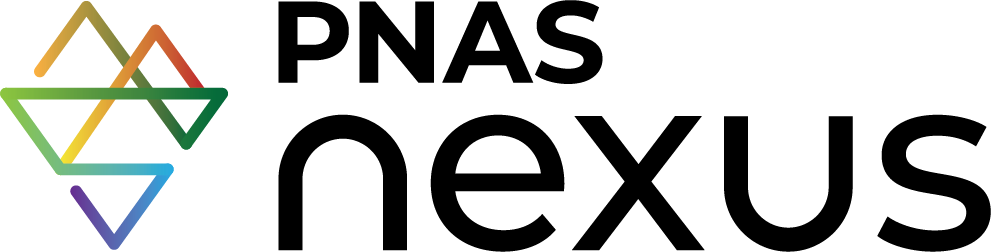
**

**Supplementary Information for**

**Capillary bifurcations mechanically dissociate clusters of tumor cells**

Angelos Vrynas¹, Aisher Chen^1^, Georgia Kontaxi^1^, Sam H. Au¹^,2^*

**Corresponding Author: Sam H. Au*

Email: s.au@imperial.ac.uk

**This PDF file includes:**

Figures S1 to S8

Tables S1 to S2

Movies S1 to S4


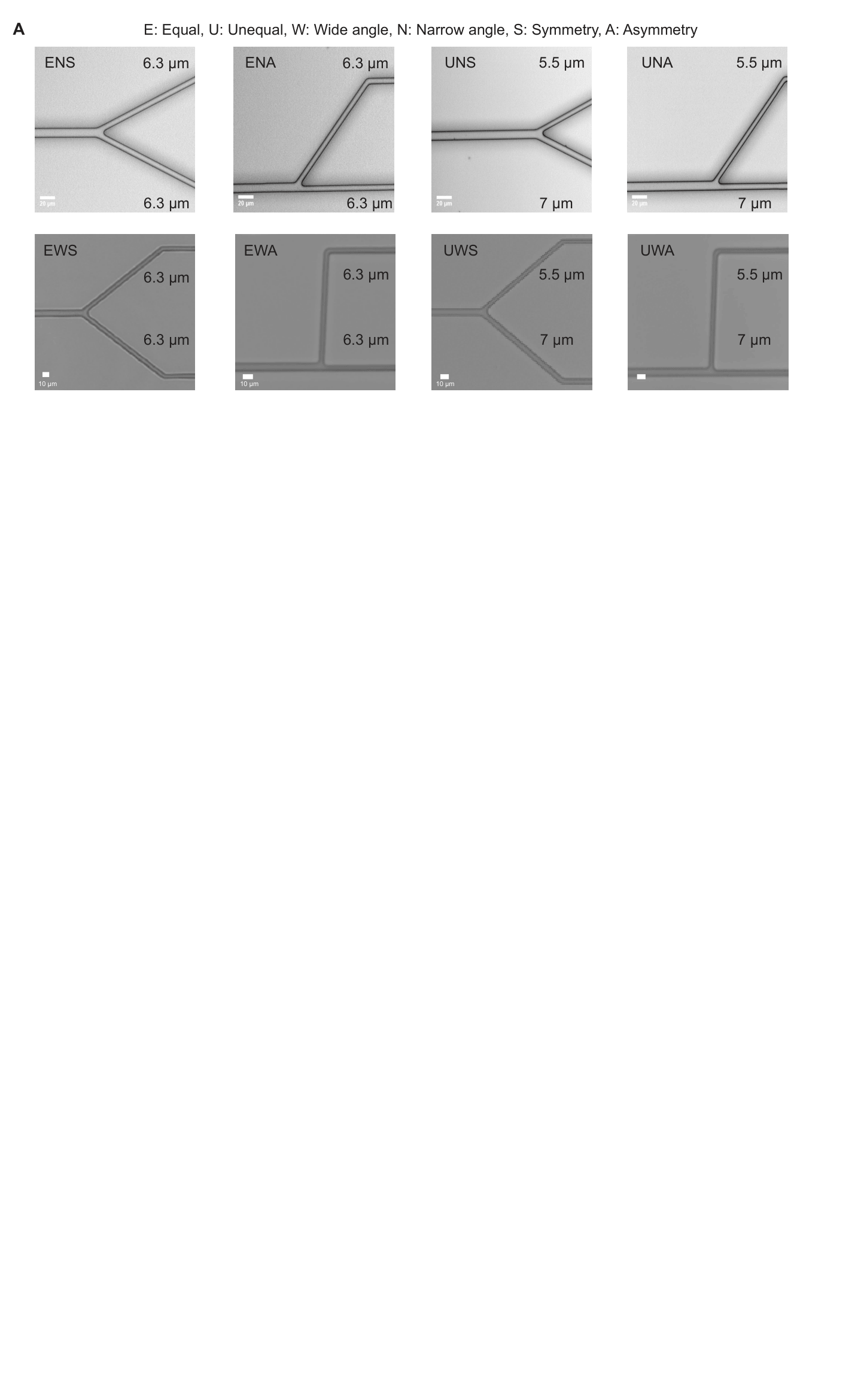


**Fig. S1. Microfluidic capillary variants.** 8 brightfield images of representative microfluidic models of capillary bifurcation variants. Scale bar: 20 μm (top) & 10 μm (bottom).


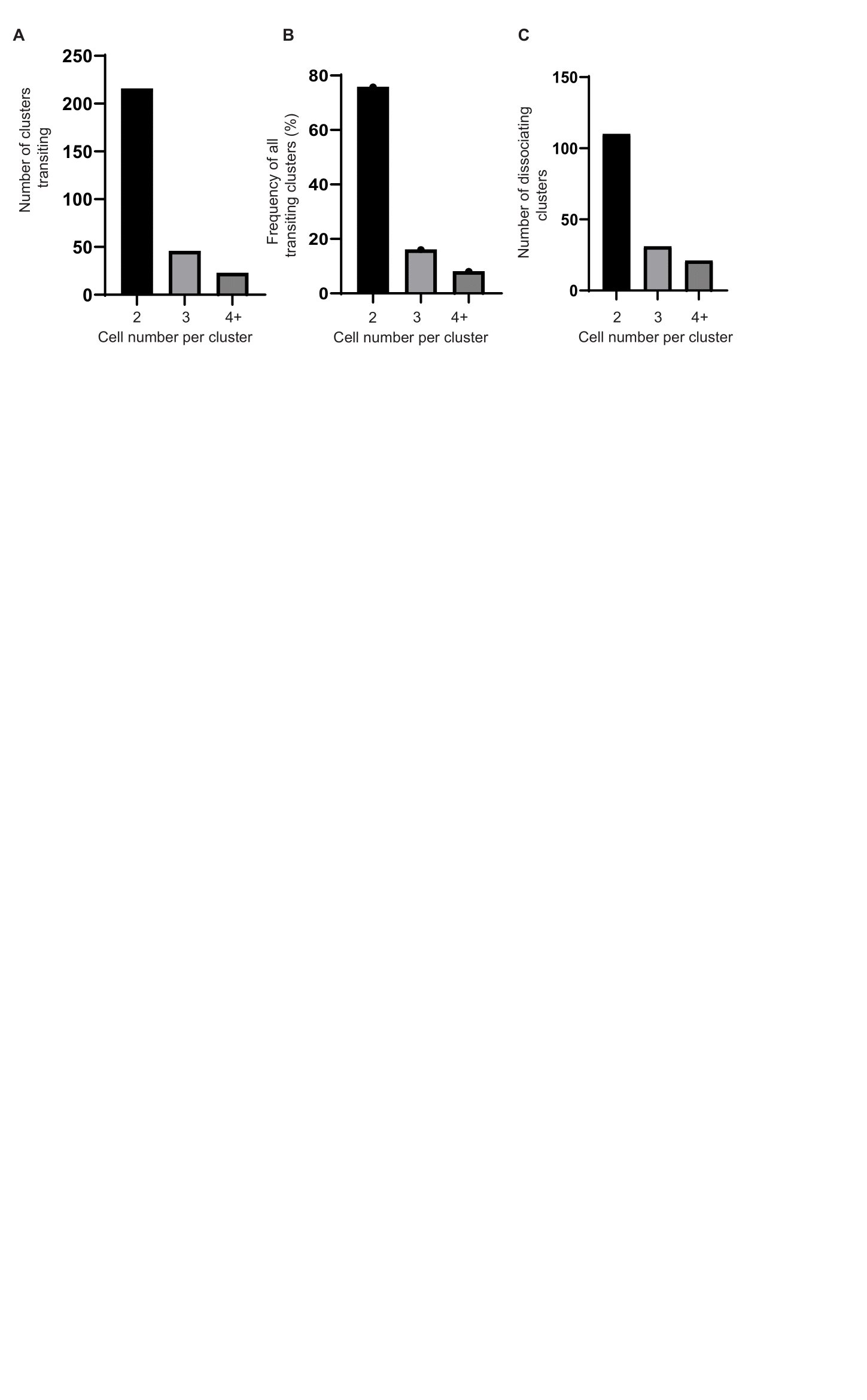


**Fig. S2. Cluster transit and dissociation in capillary bifurcations.** Quantification of clusters that successfully transited and dissociated through capillary bifurcations (EWA, EWS, UWA, UWS, UNA_5/9 & ENA_7/7): (**A**) Number of all clusters. (**B**) Percentage of doublets, triplets and quadruplets+ transiting. (**C**) Number of doublets, triplets or quadruplets+ that were observed to dissociate into small clusters and/or single cells.


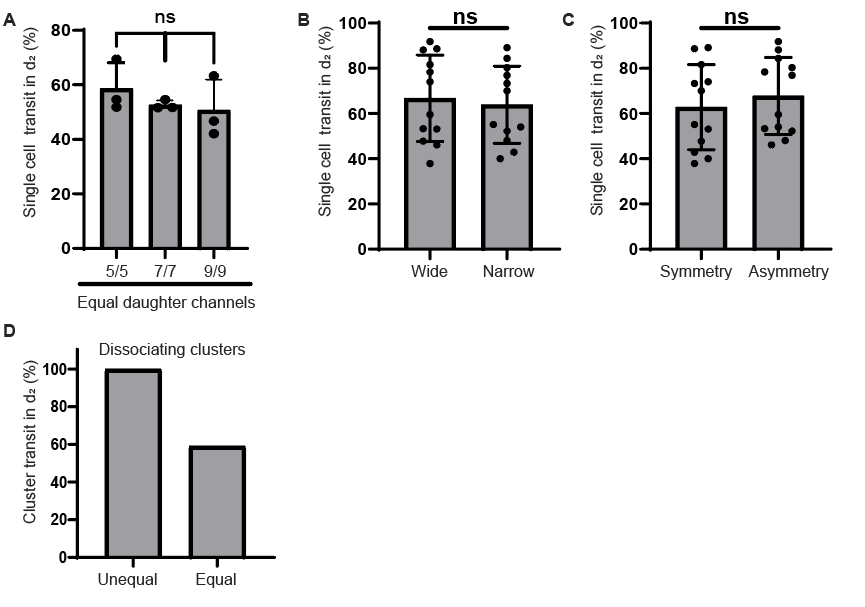


**Fig. S3. Single cells and clusters pathing in capillary bifurcations.** (**A**) Percentage of single MDA-MB231 cells recorded to move through d_2_ daughter branch channels, quantified for 3 distinct equal capillary bifurcation variants (ENA 5/5, ENA 7/7 and ENA 9/9) (n=3). (**B**) Percentage of single MDA-MB231 cells recorded to move through d_2_ daughter branch channels in wide or narrow capillary bifurcations (n=12). (**C**) Percentage of single MDA-MB231 cells recorded to move through d_2_ daughter branch channels in symmetrical or asymmetrical capillary bifurcations (n=12). (**D**) Percentage of dissociating clusters that moved through d_2_ daughter branch channels in unequal or equal capillary bifurcation variants.


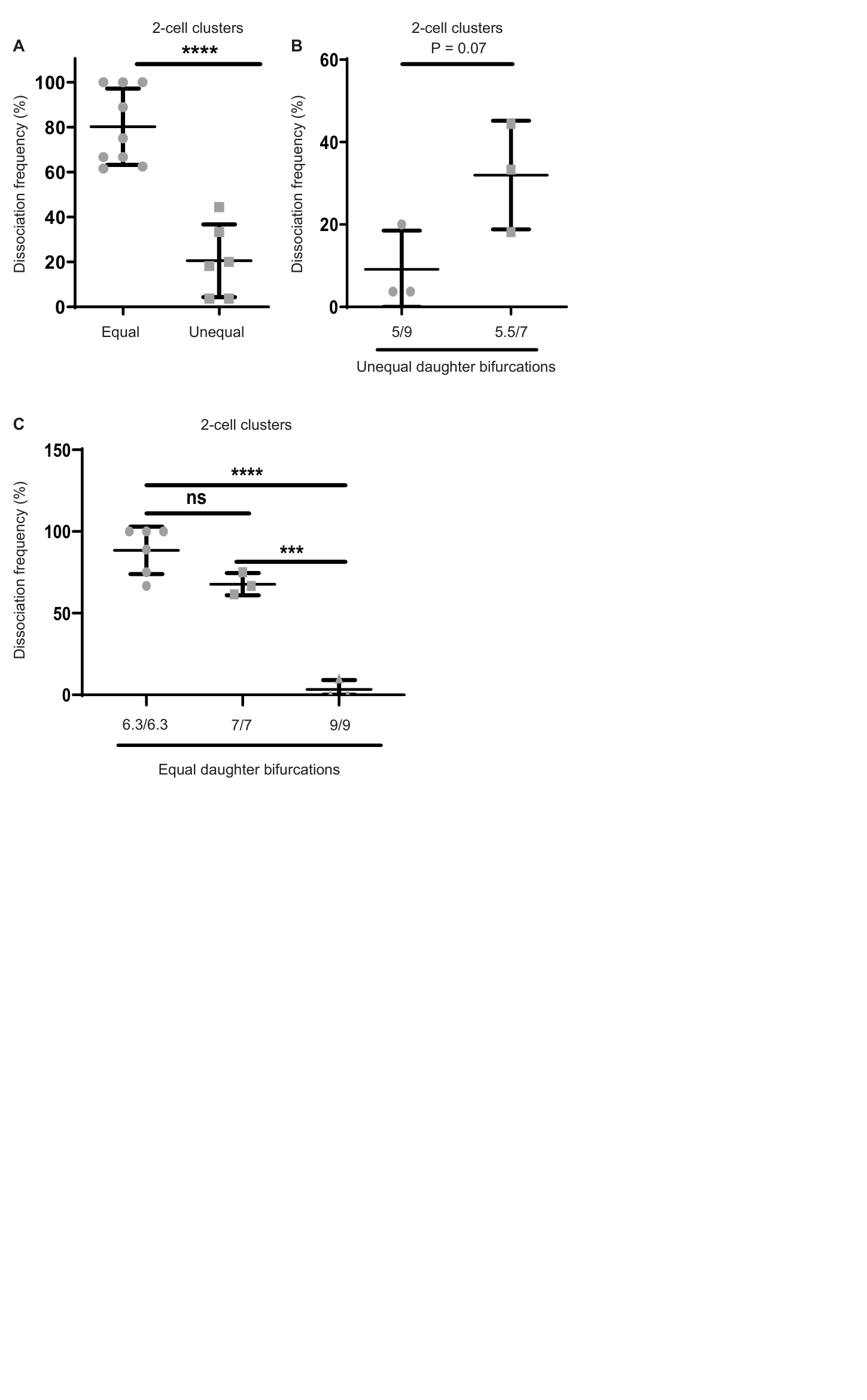


**Fig. S4. Features of capillary bifurcations and cluster volume influence doublet dissociation.** (**A**) Dissociation frequency of doublets during their transit in equal (n=9) or unequal (n=6) capillary bifurcations. (**B**) Dissociation frequency of doublets during their transit in unequal capillary bifurcations (UNA_5/9 & UNA_5.5/7) (n=3). (**C**) Dissociation frequency of doublets during their transit in equal capillary bifurcations of various sizes (n=3)).


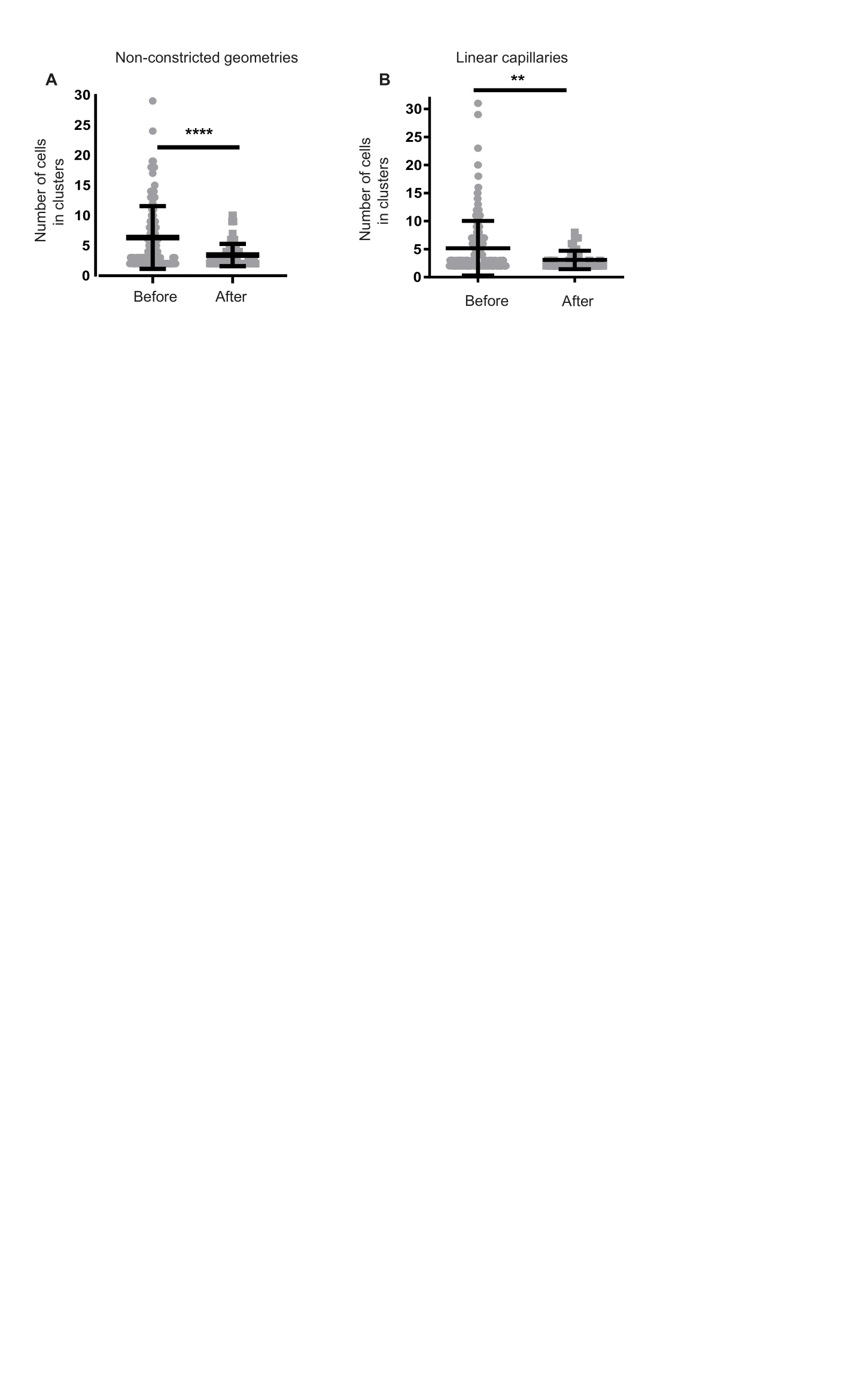


**Fig. S5. Cluster dissociation in non-constricted and linear geometries.** (**A**) Number of cells in clusters before (n=140) and post collection (n=106) from non-constricted geometries. (**B**) Number of cells in clusters before (n=164) and post collection (n=53) from linear capillaries.


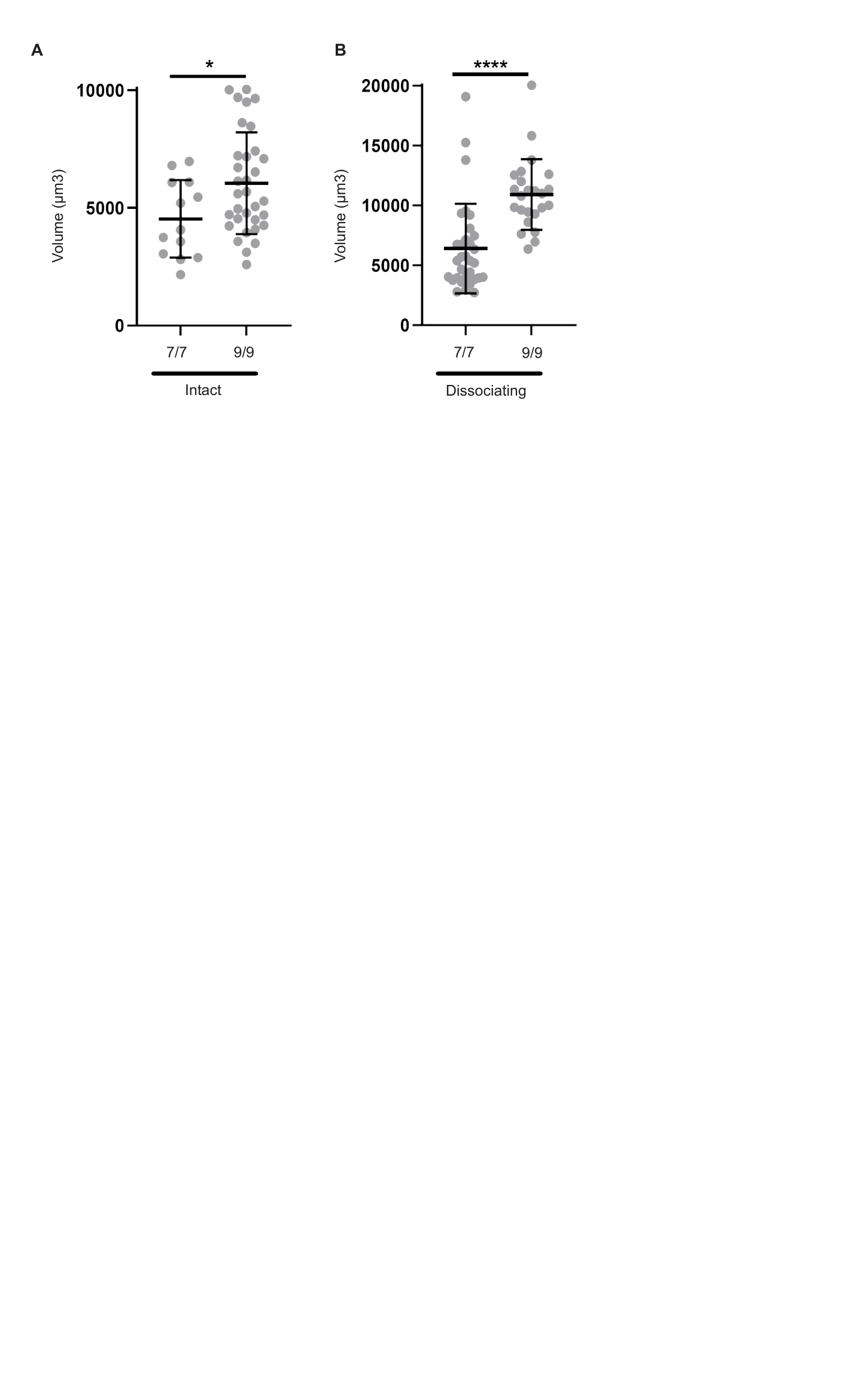


**Fig. S6. Capillary bifurcations size alters the volumetric thresholds for cluster dissociation.** (**A**) Volume of intact clusters in equal capillary bifurcations of 7 μm (n=13) or 9 μm (n=33). (**B**) Volume of dissociating clusters in equal capillary bifurcations of 7 μm (n=32) or 9 μm (n=24).


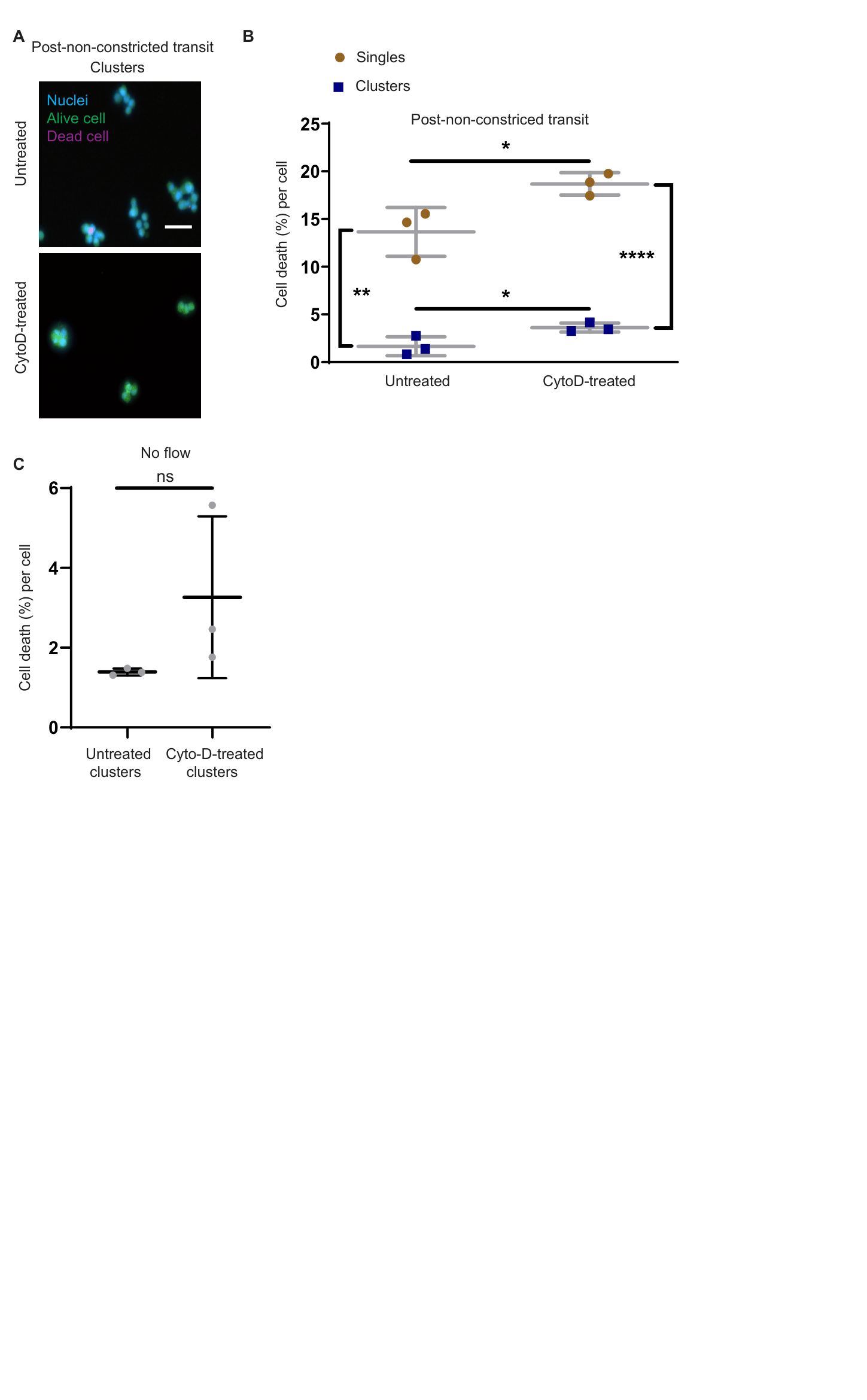


**Fig. S7. Role of Cyto-D on cell death in non-constricted devices and no flow controls.** (**A**) Fluorescent images of untreated (top) and Cyto-D-pre-treated clusters after collection from non-constricted geometries. Alive cells were stained with green cell tracker (green) and dead cells with propidium iodide (red). Nuclei was stained with Hoechst (blue). Scale bar: 50 μm. (**B**) Percentage of cell death of untreated or Cyto-D-pre-treated clusters or single cells post collection from non-constricted geometry (n=3). (**C**) Percentage of cell death of untreated or Cyto-D-pre-treated clusters under static (no flow) cultures (n=3).


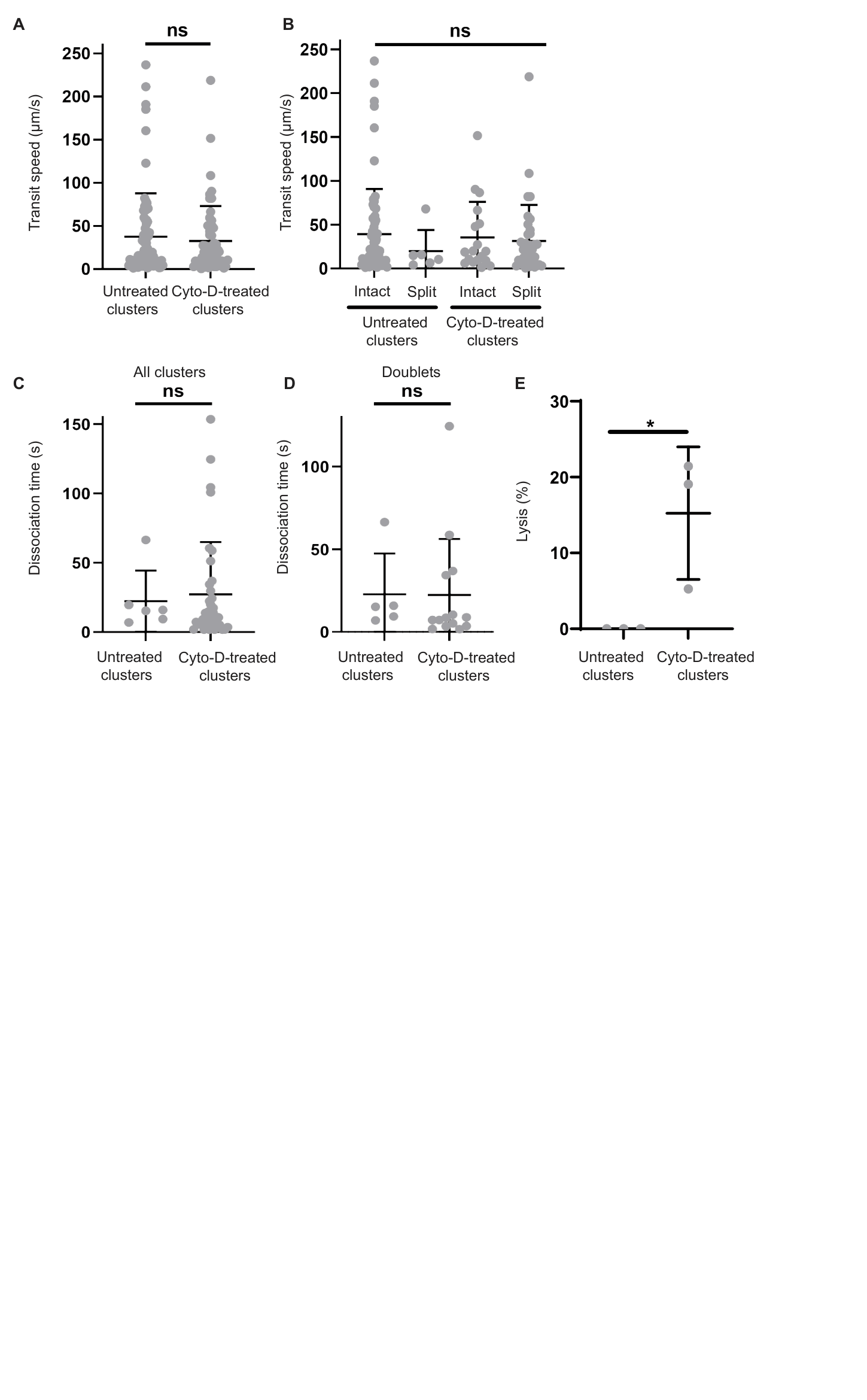


**Fig. S8. Transit speed, dissociation time and lysis of untreated or cyto-D pre-treated clusters in capillary bifurcations.** (**A**) Transit speed of untreated (n=75) or Cyto-D (54) pre-treated clusters in capillary bifurcation variant UNA_5/9 **(B)** Transit speed of untreated or Cyto-D pre-treated clusters in capillary bifurcation variant UNA_5/9, segregated in intact or dissociating (split) clusters. (**C**) Dissociation time measured from entrapment in bifurcation till dissociation, analysed for all sizes of clusters, untreated (n=6) or Cyto D treated (n=36). (**D**) Dissociation time measured from entrapment in bifurcation till dissociation, analysed for doublets only, untreated (n=5) or Cyto D treated (n=14). (**E**) Percentage of lysis during live imaging for untreated and Cyto D treated clusters (n=3). Lysis on a cluster was considered if at least one cell underwent lysis.

Table S1. Design parameters of microfluidic capillary devices: a) d1 and d2 are the effective diameter of either equal (E) or unequal (U) two daughter bifurcation channels, b) W and N represent wide (87^o^) and narrow (43.5^o^) degree angles between daughter bifurcation channels, respectively, c) Symmetry between two daughter channels represents equal angle deviation from the parent channel (S: symmetry, A: asymmetry). Non-bifurcated (linear) devices represent a straight channel without branches. Non-constricted devices represent large channels not constricting cells.

| **Name** | **Bifurcation type** | **d_1_ (μm)** | **d_2_ (μm)** |
| --- | --- | --- | --- |
| EWS_6.3/6.3 | Equal | 6.3 | 6.3 |
| EWA_6.3/6.3 | Equal | 6.3 | 6.3 |
| UWS_5.5/7 | Unequal | 5.5 | 7 |
| UWA_5.5/7 | Unequal | 5.5 | 7 |
| ENS_6.3/6.3 | Equal | 6.3 | 6.3 |
| ENA_6.3/6.3 | Equal | 6.3 | 6.3 |
| UNS_5.5/7 | Unequal | 5.5 | 7 |
| UNA_5.5/7 | Unequal | 5.5 | 7 |
| ENA_5/5 | Equal | 5 | 5 |
| ENA_7/7 | Equal | 7 | 7 |
| ENA_9/9 | Equal | 9 | 9 |
| UNA_5/7 | Unequal | 5 | 7 |
| UNA_5/9 | Unequal | 5 | 9 |
| Non-bifurcated (Linear) | N/A | N/A | N/A |
| Non-constricted | Equal | 20 | 20 |

Table S2. Comparison of expected probability of at least one dissociation event in a cluster based on observed frequency of doublet dissociation (0.51) to observed probabilities for clusters containing 3 or 4 cells.

| **Number of Cells in Cluster** | **Expected probability of dissociation** | **Observed probability of dissociation** |
| --- | --- | --- |
| 2 | 0.51 | 0.51 |
| 3 | 0.76 | 0.67 |
| 4 | 0.88 | 0.91 |

**Movie S1. Multicellular cluster dissociation in equal, narrow-angled and asymmetrical capillary bifurcation.** A 7-cell cluster dissociates in capillary bifurcation variant ENA_7/7 into 5 and 2 cells (out of frame). Cytoplasm was stained with CMFDA cell tracker (green) and nuclei with Hoechst-33342 (blue).

**Movie S2. 2-cell cluster dissociation in equal, wide-angled and asymmetrical capillary bifurcation.** A 2-cell cluster dissociates in capillary bifurcation variant EWA_6.3/6.3. Cytoplasm was stained with CMFDA cell tracker (green) and nuclei with Hoechst-33342 (blue). Note that the blue and green channels appear desynced during rapid cluster motion resulting in an artefact due to filter-switching delays but realign as the cluster slows.

**Movie S3. 3-cell cluster dissociation in 2 components in equal, wide-angled and symmetrical capillary bifurcation.** A 3-cell cluster dissociates in capillary bifurcation variant EWS_6.3/6.3 into two components; 1 single cell pathing down and a 2-cell cluster pathing up remaining intact. Shedding of a shearosome can be observed before dissociation. Cytoplasm was stained with CMFDA cell tracker (green) and nuclei with Hoechst-33342 (blue).

**Movie S4. 2-cell clusters transit in unequal, narrow-angled and asymmetrical capillary bifurcation.** A 2-cell cluster (top) moves past capillary bifurcation variant UNA_5/9 intact. A second 2-cell cluster dissociates in a parallel capillary bifurcation variant UNA_5/9. Shedding of a shearosome was observed during encounter with the bifurcation for both clusters. Cytoplasm was stained with CMFDA cell tracker (green) and nuclei with Hoechst-33342 (blue). Note that the blue and green channels appear desynced during rapid cluster motion resulting in an artefact due to filter-switching delays but realign as the cluster slows.
